# DNA methylation dataset of bovine embryonic fibroblast cells treated with epigenetic modifiers and divergent energy supply

**DOI:** 10.1016/j.dib.2022.108074

**Published:** 2022-03-22

**Authors:** Wellison J.S. Diniz, Matthew S. Crouse, Joel S. Caton, Kate J. Claycombe-Larson, Amanda K. Lindholm-Perry, Lawrence P. Reynolds, Carl R. Dahlen, Pawel P. Borowicz, Alison K. Ward

**Affiliations:** a Department of Animal Sciences, Auburn University, Auburn, AL, 36849. United States; b U.S. Meat Animal Research Center, Clay Center, USDA, ARS, NE 68933. United States; c Department of Animal Sciences, North Dakota State University, Fargo, 58108. United States; d Grand Forks Human Nutrition Research Center, USDA, ARS, ND, 58203. United States

**Keywords:** Embryonic fibroblasts, Epigenetics, Fetal programming, Methylation, One-carbon metabolites

## Abstract

Fetal programming is established early in life, likely through epigenetic mechanisms that control gene expression. Micronutrients can act as epigenetic modifiers (EM) by modulating the genome through mechanisms that include DNA methylation and post-translational modification of chromatin. Among the EM, methionine, choline, folate, and vitamin B_12_ have been suggested as key players of DNA methylation. However, the effects of supplementing these four EM, involved in the methionine folate cycle on DNA methylation, are still under investigation. This manuscript provides the genome-wide DNA methylation dataset (GSE180362) of bovine embryonic fibroblast cells exposed to different supplementation levels of glucose and methionine, choline, folate, and vitamin B_12_ (collectively named as Epigenetic Modifiers - EM). The DNA methylation was measured using *MSP-I* digestion and Reduced Representation Bisulfite Sequencing. Bioinformatics analyses included data quality control, read mapping, methylation calling, and differential methylation analyses. Supplementary file S1 and data analysis codes are within this article. To our knowledge, this is the first dataset investigating the effects of four EM in bovine embryonic fibroblast DNA methylation profiles. Furthermore, this data and its findings provide information on putative candidate genes responsive to DNA methylation due to EM supplementation.

## Specifications Table

**Table:** 

Subject	Biological Sciences
Specific subject area	Genetics: Epigenetics
Type of data	Data related to the Reduced Representation Bisulfite Sequencing (RRBS) of bovine embryonic tracheal fibroblast cells (FASTQ format) Codes Figures
How data were acquired	DNA was isolated, and bisulfite converted. Data was generated using RRBS method, including high-throughput sequencing. Paired-end sequencing with 150-bp reads was performed on the NovaSeq S Prime Illumina® platform.
Data format	Analysed, Raw
Parameters for data collection	EBTr (bovine embryonic tracheal fibroblast) cells were cultured, and treatments were arranged as a completely randomized design with two glucose levels × 3 EM levels. The control medium contained basal concentrations of folate, choline, vitamin B_12_, and methionine. Epigenetic modifiers (EM; folic acid, choline chloride, vitamin B_12_, and L-methionine) were supplemented to the media to achieve 2.5 or 5 times.
Description of data collection	DNA isolation was performed with the DNeasy Blood and Tissue Kit (Qiagen, Hilden, Germany) and quantified using the PicoGreen DNA Quantification Kit (Invitrogen). Libraries (3 samples per treatment) were prepared using the NuGEN Ovation RRBS Methyl-Seq Kit (Tecan Genomics, Redwood City, CA) and sequenced on the NovaSeq S Prime.
Data source location	Animal Nutrition Physiology Center (ANPC) – North Dakota State University. Fargo, North Dakota, USA
Data accessibility	All relevant data are within the paper and its Supplementary Information files. All sequencing data is publicly available on: **Repository name:** Gene Expression Omnibus Data identification number: GSE180362 Direct URL to data: https://www.ncbi.nlm.nih.gov/geo/query/acc.cgi?acc=GSE180362
Related research article	M.S. Crouse; J.S. Caton; K.J. Claycombe-Larson; W.J.S. Diniz; A.K. Lindholm-Perry; L.P. Reynolds; C.R. Dahlen; P.P. Borowicz; A.K. Ward. Epigenetic modifier supplementation improves mitochondrial respiration, growth rates, and alters DNA methylation of bovine embryonic fibroblast cells cultured in divergent energy supply. **Frontiers in Genetics.** doi:10.3389/fgene.2022.812764.

## Value of the Data

• This dataset provides the genome-wide DNA methylation profile of bovine embryonic fibroblast cells exposed to different levels of glucose and epigenetic modifiers.
• The dataset allows comparative methylome analysis between treatments, which could be used to identify epigenetic marks underlying diet-induced fetal programming.
• Diet-induced epigenome profile sheds light on the role of epigenetic modifiers in fetal programming.

## 1 Data Description

We performed a genome-wide DNA methylation analysis of bovine embryonic fibroblast cells to investigate the effects of glucose and epigenetic modifiers (EM) supplementation on cell epigenetic programming [1]. Herein, we describe the methylome datasets generated using Reduced Representation Bisulfite Sequencing (RRBS). Data was generated from Bovine Embryonic Tracheal fibroblast cells treated with two glucose levels and 3 different concentrations of EM (3 samples per treatment). Table 1 shows a summary of the metadata, sample description, mapping statistics per sample, the experimental design, and the number of replicates per treatment as described elsewhere [1]. The **(1)** raw paired-end RRBS reads from 18 samples; **(2)** the metadata; and **(3)** the normalized DNA methylation levels are publicly available on the GEO database (GEO accession ID GSE180362); **(4)** Supplementary File S1 is the code used for DNA methylation data analysis as described in the methods section.

**Table 1:** Summary of the RRBS data from bovine embryonic fibroblast cells treated with epigenetic modifiers and divergent levels of glucose.

Acession number	File name	T	R	% mCpG	% Aligned	M Seqs
GSM5461479	EBTr_High_Glc_2-5X_1_S16	H_2.5X	1	54.0%	35.2%	21.4
GSM5461480	EBTr_High_Glc_2-5X_2_S17	H_2.5X	2	55.7%	34.6%	10.2
GSM5461481	EBTr_High_Glc_2-5X_3_S18	H_2.5X	3	55.4%	34.6%	7
GSM5461482	EBTr_High_Glc_5-0X_1_S19	H_5.0X	1	55.3%	35.4%	8.4
GSM5461483	EBTr_High_Glc_5-0X_2_S20	H_5.0X	2	54.9%	35.1%	7.2
GSM5461484	EBTr_High_Glc_5-0X_3_S21	H_5.0X	3	54.8%	35.1%	16.4
GSM5461485	EBTr_High_Glc_CON_1_S13	H_CON	1	55.1%	35.2%	18
GSM5461486	EBTr_High_Glc_CON_2_S14	H_CON	2	54.8%	33.2%	13.7
GSM5461487	EBTr_High_Glc_CON_3_S15	H_CON	3	54.7%	36.0%	18.8
GSM5461488	EBTr_Low_Glc_2_5X_1_S4	L_2.5X	1	55.6%	35.4%	10.1
GSM5461489	EBTr_Low_Glc_2_5X_2_S5	L_2.5X	2	55.4%	35.4%	20.2
GSM5461490	EBTr_Low_Glc_2_5X_3_S6	L_2.5X	3	55.0%	35.8%	21.6
GSM5461491	EBTr_Low_Glc_5_0X_1_S7	L_5.0X	1	55.6%	35.8%	29.5
GSM5461492	EBTr_Low_Glc_5_0X_2_S8	L_5.0X	2	55.6%	34.6%	18.9
GSM5461493	EBTr_Low_Glc_5_0X_3_S9	L_5.0X	3	55.8%	36.1%	14.4
GSM5461494	EBTr_Low_Glc_CON_1_S1	L_CON	1	55.1%	34.9%	15.3
GSM5461495	EBTr_Low_Glc_CON_2_S2	L_CON	2	55.8%	35.7%	5.8
GSM5461496	EBTr_Low_Glc_CON_3_S3	L_CON	3	55.6%	35.1%	4.3

**T:** Treatments. High (**H**) or low (**L**) levels of glucose, arranged with control, 2.5, or 5 times epigenetic modifier levels. **R**: Replicates. % **mCpG**: Percentage of methylated cytosines, **%Aligned**: Percentage of unique mapped reads to the reference genome. **M seqs:** Number of clean reads per sample in million.

**Fig. 1:**
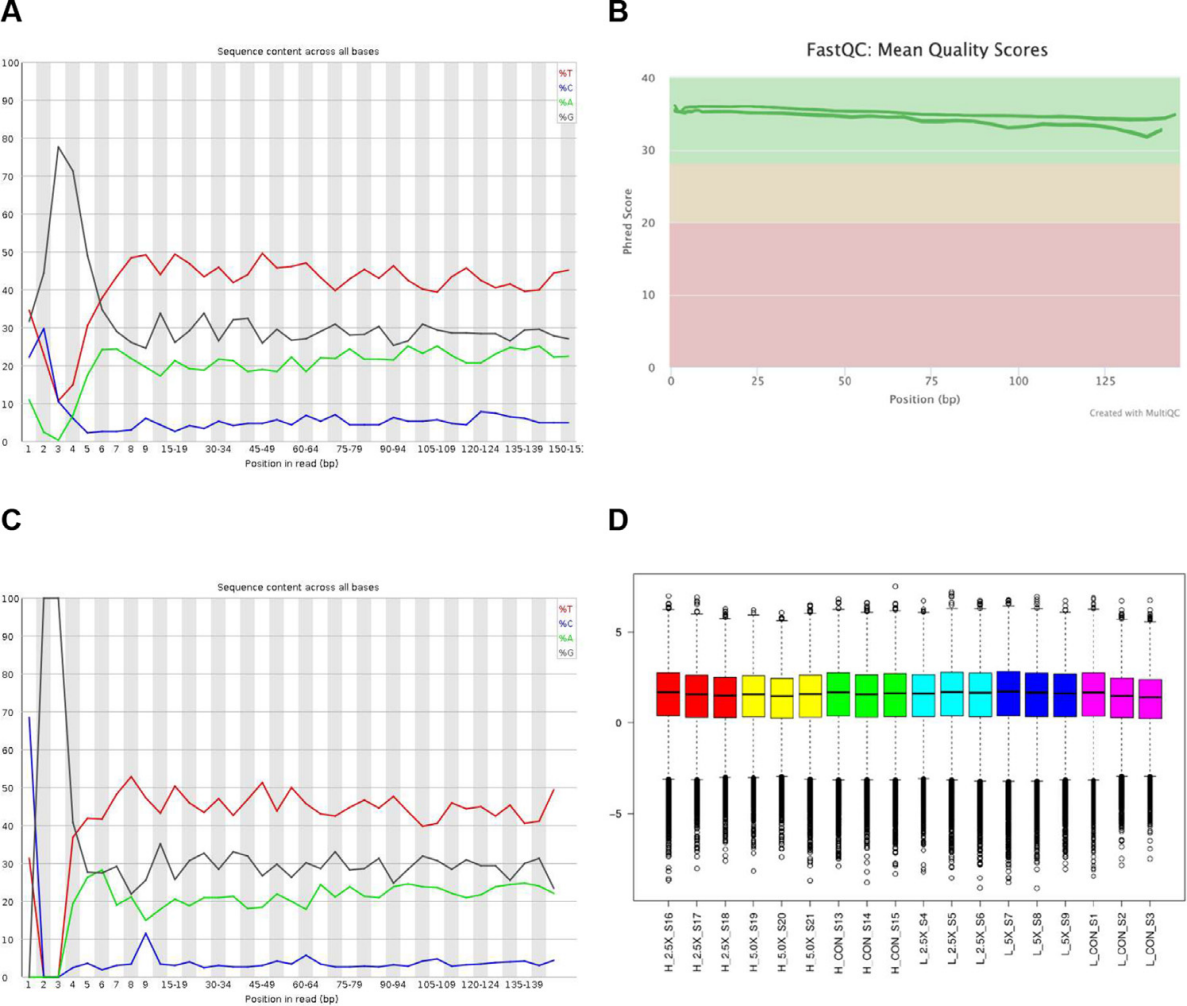
Overview of RRBS data from bovine embryonic fibroblast cells treated with epigenetic modifiers and divergent levels of glucose. (A) Per base sequence content; (B) Overall Phred score; (C) Per base sequence content after NuGEN diversity's adapter trimming; (D) Distributions of RRBS methylation data after normalization (M-values) for 18 samples (3 samples per treatment). The M-values and sample IDs are represented in the *Y-* and *x-axis*, respectively.

The raw data is in the FASTQC format, and an average of 14.5 M reads was generated for each sample. Successful sequencing, adapter and diversity trimming are shown in Fig. 1A. Furthermore, overall sequence read quality assessed by Phred score was > 30 (Fig. 1B). As expected, after the diversity adapters were filtered out, we can see the *MspI* site signature – YGG, at the 5’ end (Fig. 1C). A total of 261.2 M reads were kept after quality control and, on average, 35.18% were uniquely mapped to the reference genome. The representation of the normalized DNA methylation levels for each sample retrieved from Bismarck are presented in Fig. 1D. The boxplot shows fairly consistent medians across the 18 samples in the dataset. The normalized methylation levels for each sample are available on GEO (GSE180362) database.

## 2 Experimental Design, Materials and Methods

### 2.1 Cell culture and treatments

The Bovine Embryonic Tracheal Fibroblast cell lines (EBTr; NBL-4; ATCC CCL-44) were purchased from the American Type Culture Collection (Manassas, VA). Cell culture was performed into T-75 culture flasks with Eagle's Minimum Essential Medium (EMEM: Sigma, St. Louis, MO) (2) supplemented with 10% fetal bovine serum (Thermo Fisher Scientific, Waltham, MA), 1% penicillin-streptomycin (Thermo Fisher Scientific) and 0.11 g/L Na pyruvate (Sigma). Culture conditions were as follows: 37 °C, 5% CO_2_ incubator, and passaged at approximately 90% confluence.

The study was arranged as a completely randomized design with two glucose levels × 3 EM levels. Control medium contained basal concentrations of folate (0.001 g/L), choline (0.001 g/L), vitamin B_12_ (4 µg/L), and methionine (0.015 g/L). The treatment medium was composed of glucose [1 g/L (Low) or 4.5 g/L (High)] added as D-glucose (Sigma). Furthermore, EM (folic acid, choline chloride, vitamin B_12_, and L-methionine) was supplemented to achieve 2.5 or 5 times of the concentrations in the control medium, except for methionine, which was limited at 2X across all supplemented treatments to prevent toxicity.

### 2.2 DNA isolation, library preparation and sequencing

Cells were harvested once confluency reached 90%, and cell pellets (1 × 10^6^) were frozen in cryovials (n = 3 samples/treatment). The DNA isolation protocol followed the DNeasy Blood and Tissue Kit (Qiagen, Hilden, Germany) guidelines. Further, DNA quality control and quantification were performed using the PicoGreen DNA Quantification Kit (Invitrogen). The NuGEN Ovation RRBS Methyl-Seq Kit (Tecan Genomics, Redwood City, CA) was used for library preparation from 20 ng/µL of DNA. After bisulfite conversion and PCR amplification, sequencing (n = 18) was performed with NovaSeq S Prime in the 150 paired-end reads mode (Illumina, San Diego, CA). All the molecular analyses and sequencing were performed by the University of Minnesota Genomics Center (Minneapolis, MN, USA).

### 2.3 Data analysis

After sequencing, raw data quality control was performed using the FastQC v0.11.8 and MultiQC v1.9 software. Adapter trimming and low-quality bases (Phred score < 20) were filtered out using Cutadapt v.2.10. Then, NuGEN's diversity adaptors were trimmed by a custom python script – *trimRRBSdiversityAdaptCustomers.py,* provided by NuGEN. The script removes any reads that do not contain a *MspI* site signature YGG at the 5’ end.

Trimmed reads were mapped to the bovine reference genome (UCSC - bosTau8, Illumina IGenomes) using Bismarck with Bowtie2 within the nf-core/methylseq pipeline v1.5 [2]. Only cytosines in a CpG context were retrieved and analyzed. Reads containing CpGs with more than 99.9th percentile coverage and less than ten counts in every sample were filtered out to avoid biases due to varying sequencing depth.

Differentially methylated cytosines (DMC) were identified by pair-wise comparison using edgeR v.3.24.3 [3], and considered significant for each of the contrasts when the *P*-value cutoff ≤ 0.01 [3]. Methylation levels (M-value) were normalized based on the following equation: M = log_2_ {(Me + α)/(Un + α)}, where Me and Un are the methylated and unmethylated intensities and α is some suitable offset to avoid taking logarithms of zero [3]. The codes describing the aforementioned analysis are within Supplementary File S1.

## Ethics Statement

Does not apply.

## CRediT authorship contribution statement

**Wellison J.S. Diniz:** Formal analysis, Writing – original draft, Writing – review & editing. **Matthew S. Crouse:** Conceptualization, Methodology, Funding acquisition, Writing – review & editing. **Joel S. Caton:** Conceptualization, Methodology, Writing – review & editing. **Kate J. Claycombe-Larson:** Writing – review & editing. **Amanda K. Lindholm-Perry:** Writing – review & editing. **Lawrence P. Reynolds:** Methodology, Writing – review & editing. **Carl R. Dahlen:** Methodology, Writing – review & editing. **Pawel P. Borowicz:** Methodology, Writing – review & editing. **Alison K. Ward:** Conceptualization, Methodology, Supervision, Writing – review & editing.

## Declaration of Competing Interest

The authors declare that they have no known competing financial interests or personal relationships which have or could be perceived to have influenced the work reported in this article.

## Data Availability

RRBS DNA methylation data of bovine embryonic fibroblast cells (Original data) (GEO).

## Appendix Supplementary materials

Image, application 1
