# Supplementary material for: DNA methylation dataset of bovine embryonic fibroblast cells treated with epigenetic modifiers and divergent energy supply
Source: Data Brief. 2022 Mar 22;42:108074. doi: 10.1016/j.dib.2022.108074 (PMC8980535; doi:10.1016/j.dib.2022.108074)
Supplement: Supplementary file 1 [file mmc1.zip › DIB_Code.html]

DNA methylation dataset of bovine embryonic fibroblast cells


# DNA methylation dataset of bovine embryonic fibroblast cells

> By **W.J.S. Diniz**

## Data availability

Data is available in GEO under the accession number GSE180362.

# Software used

| Software | Version | URL |
| --- | --- | --- |
| FastQC | 0.11.8 | http://www.bioinformatics.babraham.ac.uk/projects/fastqc/ |
| MultiQC | 1.9 | https://multiqc.info/ |
| Cutadapt | 2.10 | https://cutadapt.readthedocs.io/ |
| NuGEN Ovation RRBS | - | https://github.com/nugentechnologies/NuMetRRBS |
| methylseq | 1.5 | https://nf-co.re/methylseq/1.5 |
| edgeR | 3.24.3 | https://bioconductor.org/packages/release/bioc/html/edgeR.html |
|  |  |  |

```
This part of the code was run in HPC cluster (CCAST/Thunder - NDSU) with a .PBS script
```

# **Step 1**

**Reads Quality Control**

## FastQC

```
INPUTDIR=/path/to/fastq/files/

OUTDIR=/path/to/a/directory/to/store/the/results/


fastqc -t $NCPUS -o $OUTDIR $INPUTDIR/*.fastq.gz
```

## MultiQC

```
DIR=/path/to/directory/with/the/results/from/fastQC/

OUTDIR=/path/to/a/directory/to/store/the/results/

multiqc $DIR -o $OUTDIR
```

## Cutadapt

```
#Set directories
IN_DIR="/path/to/fastq/files/" #make sure it is between " " 
OUT_DIR="/path/to/a/directory/to/store/the/results/"

# Set the adapter sequences
ADAPTER1="AGATCGGAAGAGC"
ADAPTER2="AAATCAAAAAAAC"

# Find the files to process. Start with R1 and build R2 from that
R1=($(find "${IN_DIR}" -type f -name '*_R1_001.fastq.gz' -exec basename {} \; | sort -V | sed -e 's/_R1_001.fastq.gz//g'))

for read1 in ${R1[@]}
do
   cutadapt -q 20 -m 15 -a ${ADAPTER1} -A ${ADAPTER2} -o ${OUT_DIR}/${read1}_R1.trimmed.fastq.gz -p ${OUT_DIR}/${read1}_R2.trimmed.fastq.gz ${IN_DIR}/${read1}_R1_001.fastq.gz ${IN_DIR}/${read1}_R2_001.fastq.gz

done
```

## Nugen Diversity Trimming

make sure you download the python scripts and follow the Analysis Guide for NuGEN Ovation RRBS Methyl-Seq

```
IN_DIR="/path/to/fastq/files/after/removing/the/adapters/"

R1=($(find "${IN_DIR}" -type f -name '*_R1.trimmed.fastq.gz' -exec basename {} \; | sort -V | sed -e 's/_R1.trimmed.fastq.gz//g'))

for read1 in ${R1[@]}
do

python /path/to/directory/with/the/nugen/python/scripts/trimRRBSdiversityAdaptCustomers.py \
-1 ${IN_DIR}/${read1}_R1.trimmed.fastq.gz -2 ${IN_DIR}/${read1}_R2.trimmed.fastq.gz

done
```

**Repeat FastQc and MultiQC** using the trimmed files.

# **Step 2**

## Read mapping and Cytosine methylation

Please follow the instructions from nf-core to install and run nextflow pipelines. Parameters, description and usage docs for methylseq are described here.

We used:  
\* Bismarck as aligmnet tool  
\* bosTau8 genome from Illumina IGenomes

```
nextflow run nf-core/methylseq \
-with-singularity methylseq_latest.sif \
--reads '/path/to/trimmed/reads//*_{R1,R2}.trim.fq.gz' \
--genome bosTau8 --skip_trimming --outdir '/path/to/results/directory' \
--skip_deduplication --max_memory '40.GB' --max_time '5.h' --max_cpus 10
```

# **Step 3**

**edgeR**

Differential methylation analysis of reduced representation bisulfite sequencing data was performed following the edgeR tutorial available here.

Methylation level was normalized based on the M-value, which is defined as M = log2 {(Me + α)/(Un + α)} (**see edgeR tutorial**). M contains the empirical logit methylation level for each CpG site in each sample.

```
M <- log2(Me + 2) - log2(Un + 2)
```

```
#Barplot methylation distribution between samples

#colored by groups 

#Color vector based on the number of samples 
col <- rainbow(nrow(targets))

#getting the treatment groups

sl.colors<- as.data.frame (targets$Condition)

#matching samples to groups

row.names(sl.colors)<-row.names(targets)

group.plot <- as.character(targets$Condition)

sl.colors[,1] <- as.character(sl.colors[,1])

names(col) <- group.plot

col.sl <- col[sl.colors[,1]]

par(mar = c(10,4,4,2))
par(las=2)
par (cex=1)
par(las=2)
boxplot(M, col=col.sl, main="M-values")
```
